# Dataset on the synthesis and physicochemical characterization of blank and curcumin encapsulated sericin nanoparticles obtained from *Philosamia ricini* silkworm cocoons

**DOI:** 10.1016/j.dib.2019.104359

**Published:** 2019-08-22

**Authors:** Devangana Bhuyan, George Warren Greene, Ratul Kumar Das

**Affiliations:** a TERI-Deakin Nanobiotechnology Centre, The Energy and Resources Institute (TERI), Gual Pahari, Haryana, 122 001, India; b Institute for Frontier Materials (IFM), Deakin University, Burwood, Melbourne, 3125, Australia

**Keywords:** Sericin, Curcumin, Sericin nanoparticles, Encapsulation efficiency, Loading capacity

## Abstract

The dataset presents the synthesis and physicochemical characterization of blank and curcumin encapsulated sericin protein nanoparticles obtained from *Philosamia ricini* (also known as Ahimsa silk or Peace silk or Eri). Reports on application of sericin protein obtained from *P. ricini* are scanty at best. Sericin was extracted from the cocoons by high temperature and high pressure method. Synthesis of sericin nanoparticles was carried out by desolvation method using acetone as the desolvating agent. Curcumin was used as a hydrophobic model drug and was encapsulated into the sericin nanoparticles. Physicochemical characterization of the blank and curcumin encapsulated sericin nanoparticles were carried out by different instrumental analyses. The size and surface charges of sericin nanoparticles changed with the variation of applied sericin concentration. Encapsulation efficiency and loading capacity of the encapsulated sericin nanoparticles showed variation with curcumin concentration. The obtained data indicated the applicative potentials of sericin protein extracted from *Philosamia ricini* silkworm cocoons.

**Table:** Specifications table

Subject area	Seribiotechnology
More specific subject area	Drug delivery, Food preservation, Antimicrobial activity
Type of data	Table, image, graph, figure
How data was acquired	UV–Vis spectrophotometer: Shimadzu UV2450 Zetasizer: Malvern ZEN3690 SEM: Zeiss Evo MA 10 FTIR: Thermo Scientific Nicolet 6700 FT-IR
Data format	Raw and graphs
Experimental factors	*Philosamia ricini* (Eri) cocoons were cleaned and degummed by the high temperature and high pressure method followed by filtration, lyophilization, desolvation and encapsulation
Experimental features	Synthesis and physicochemical characterization of sericin nanoparticles for encapsulation of hydrophobic model drug ‘curcumin’ into a hydrophilic system by desolvation method
Data source location	TERI-Deakin Nanobiotechnology Centre, The Energy and Resources Institute, TERI-Gram, Gual Pahadi, Haryana, India
Data accessibility	Data provided within this article

**Table:** 

**Value of the data** • Sericin is a fibrous hydrophilic protein with intermittent hydrophobic domains, holding tremendous potential for fundamental research [2]. The data on physicochemical characterization of the blank and curcumin encapsulated sericin nanoparticles presented here could be experimented with other hydrophobic drug molecules for application in the fields of drug delivery, food preservation and antimicrobial activity • The present dataset could be useful for design and development of sericin or other hydrophilic protein based nano-carriers with applicative potentials in drug delivery, food preservation, and nutraceuticals, among others • Further studies could shed light on the β-sheet transition of Eri sericin as well as the type of interaction occurring between the sericin and curcumin molecules • Sericin being soluble in water and reportedly to have a strong emulsifying property, the present dataset could provide the fundamentals for preparation of emulsions of oil-in-water type with wide applications

## 1 Data

**Fig. 1:**
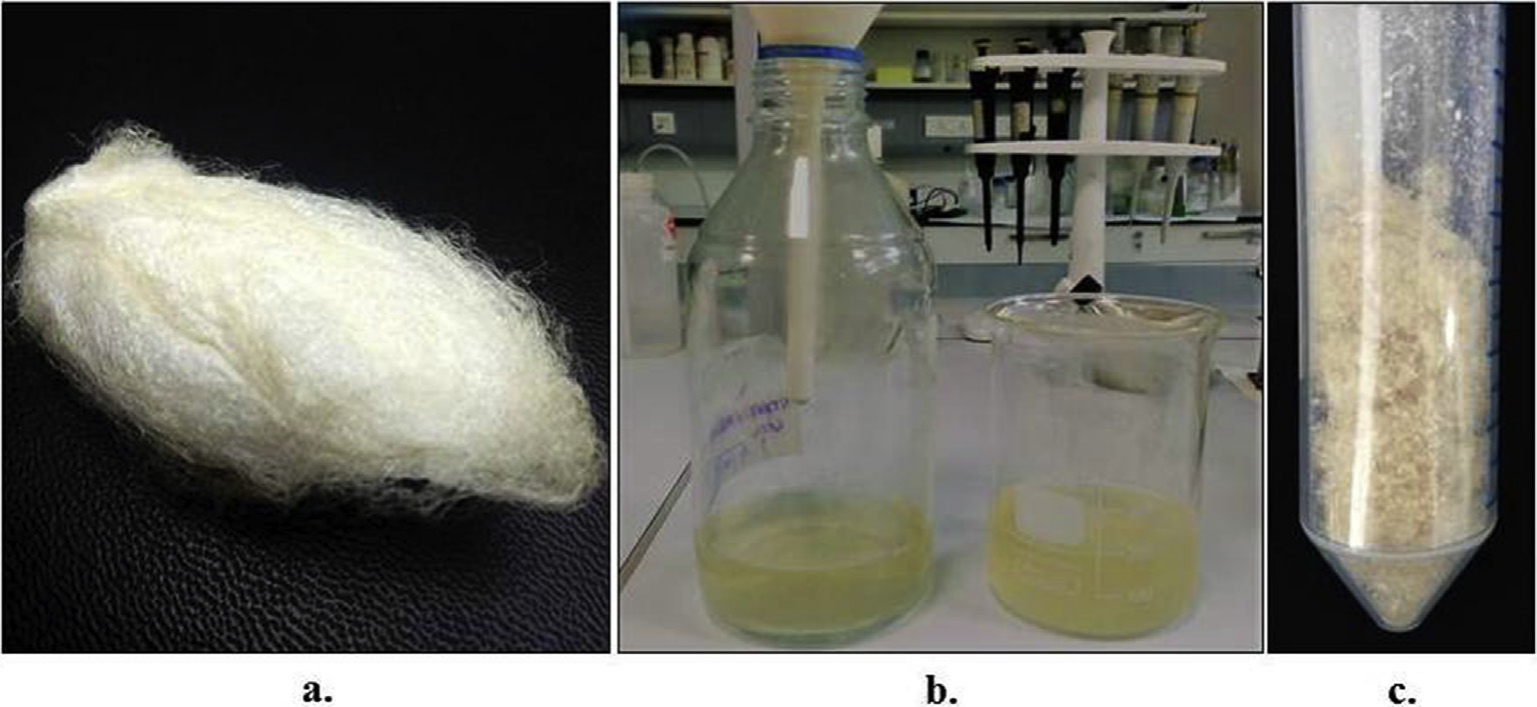
Sericin extraction from silk cocoons. a. Silk cocoon of *Philosamia ricini* silkworm. b. Extracted sericin. c. Lyophilized sericin.

**Fig. 2:**
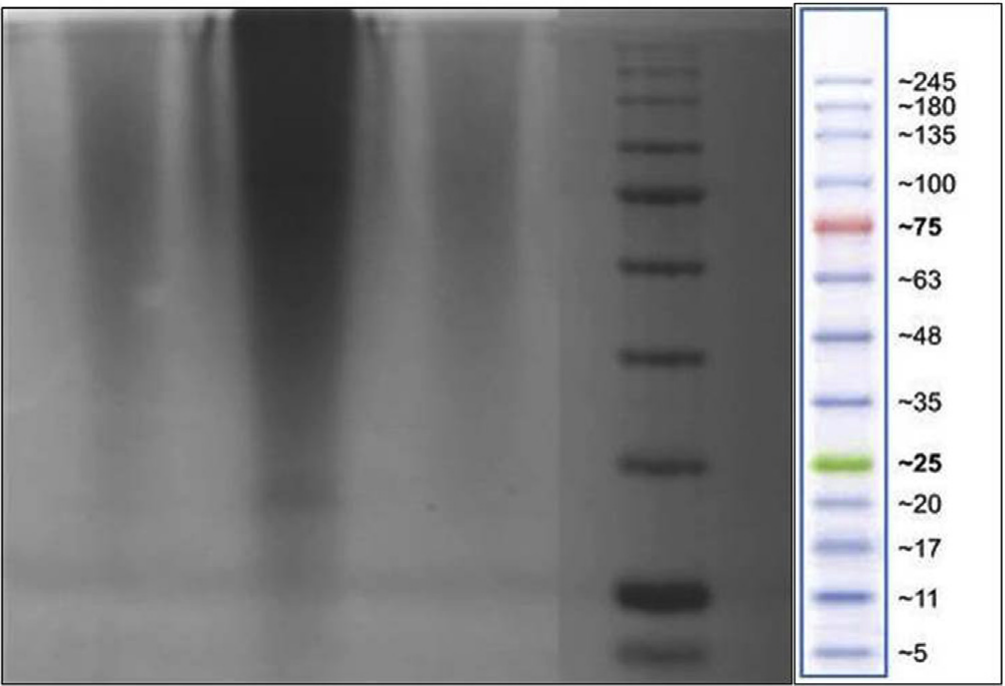
SDS-PAGE gel image showing bands of sericin and protein marker.

**Fig. 3:**
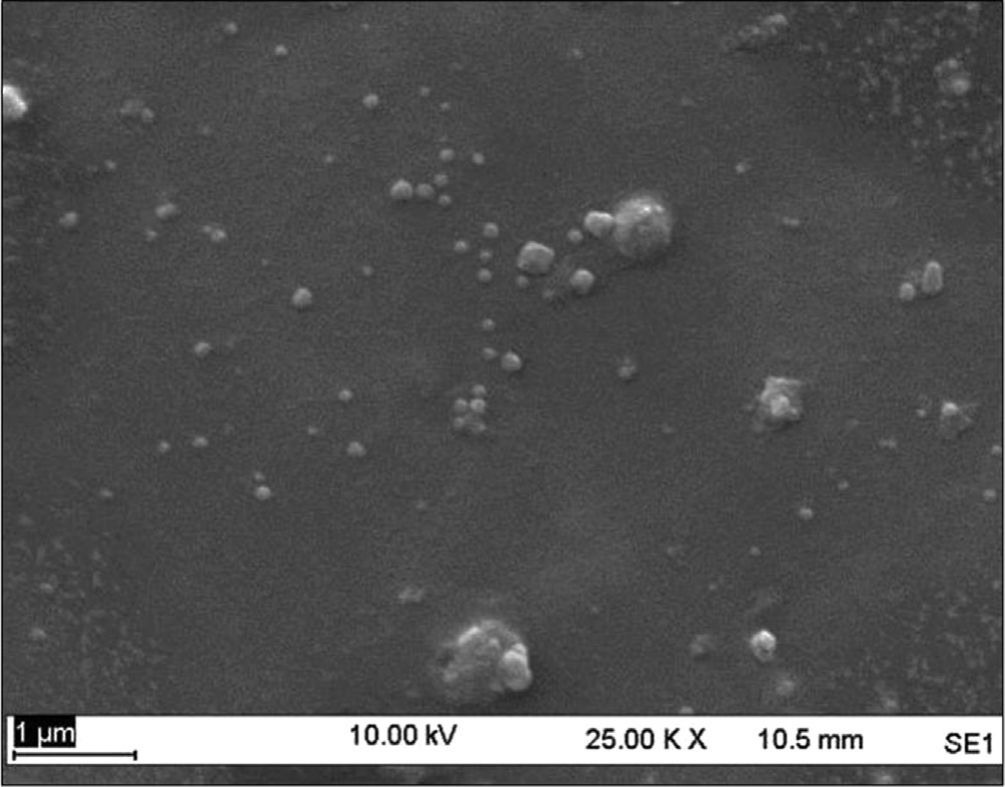
SEM image of sericin nanoparticles at sericin concentration of 0.5 mg mL^−1^.

**Fig. 4:**
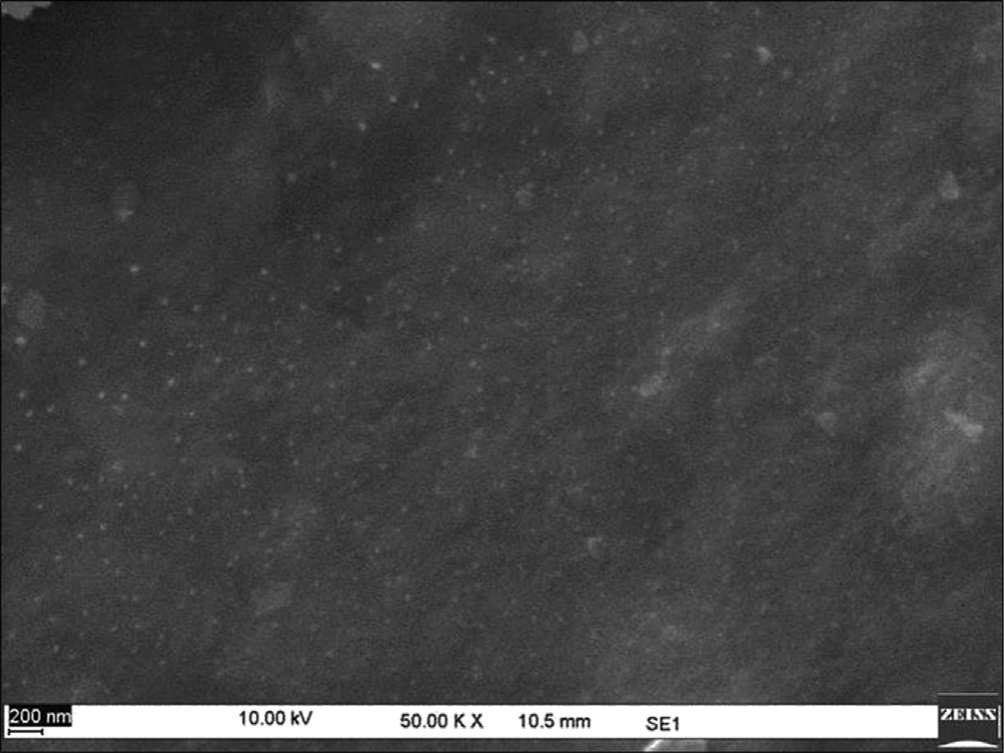
SEM image of sericin nanoparticles at sericin concentration of 1.0 mg mL^−1^.

**Fig. 5:**
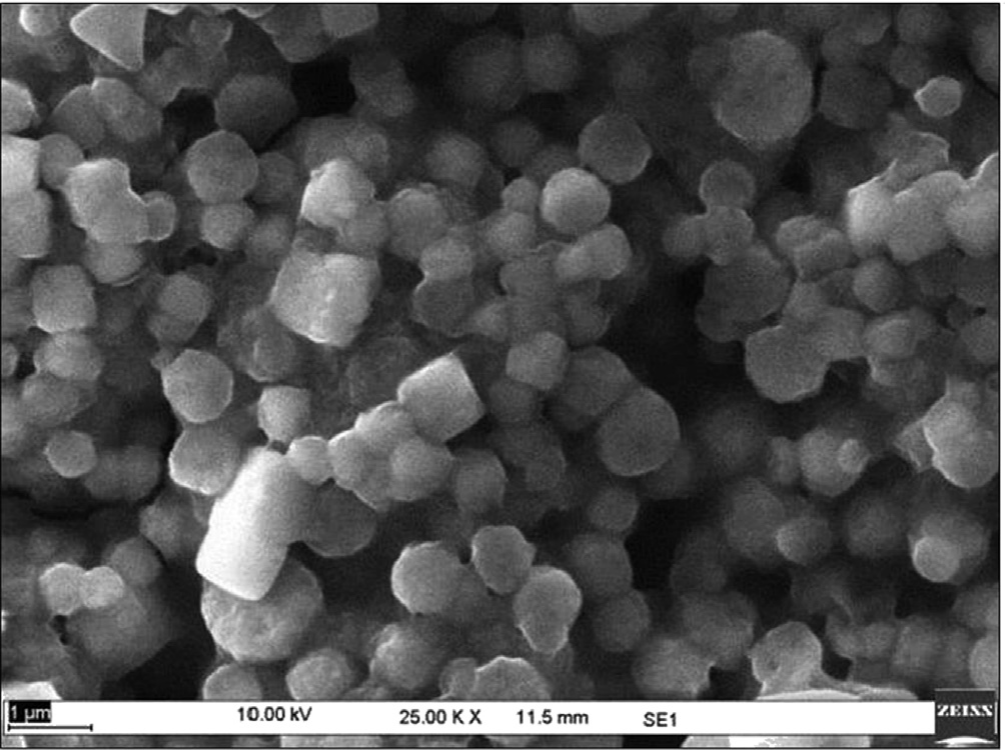
SEM image of sericin nanoparticles at sericin concentration of 1.5 mg mL^−1^.

**Fig. 6:**
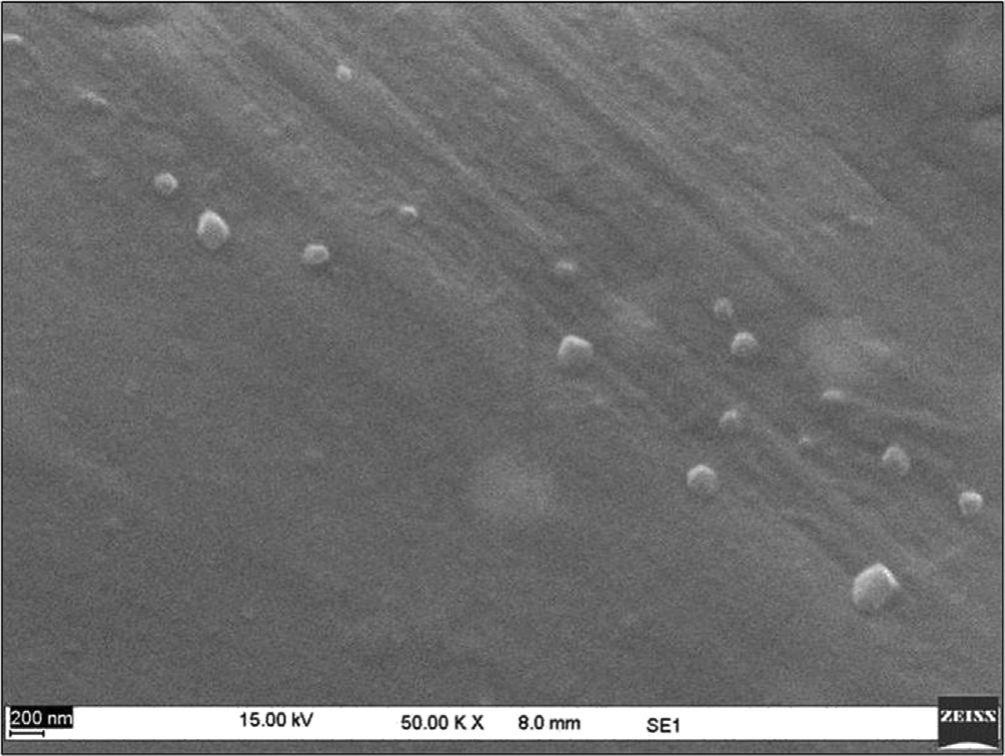
SEM image of curcumin encapsulated sericin nanoparticles at 200 μM curcumin concentration.

**Fig. 7:**
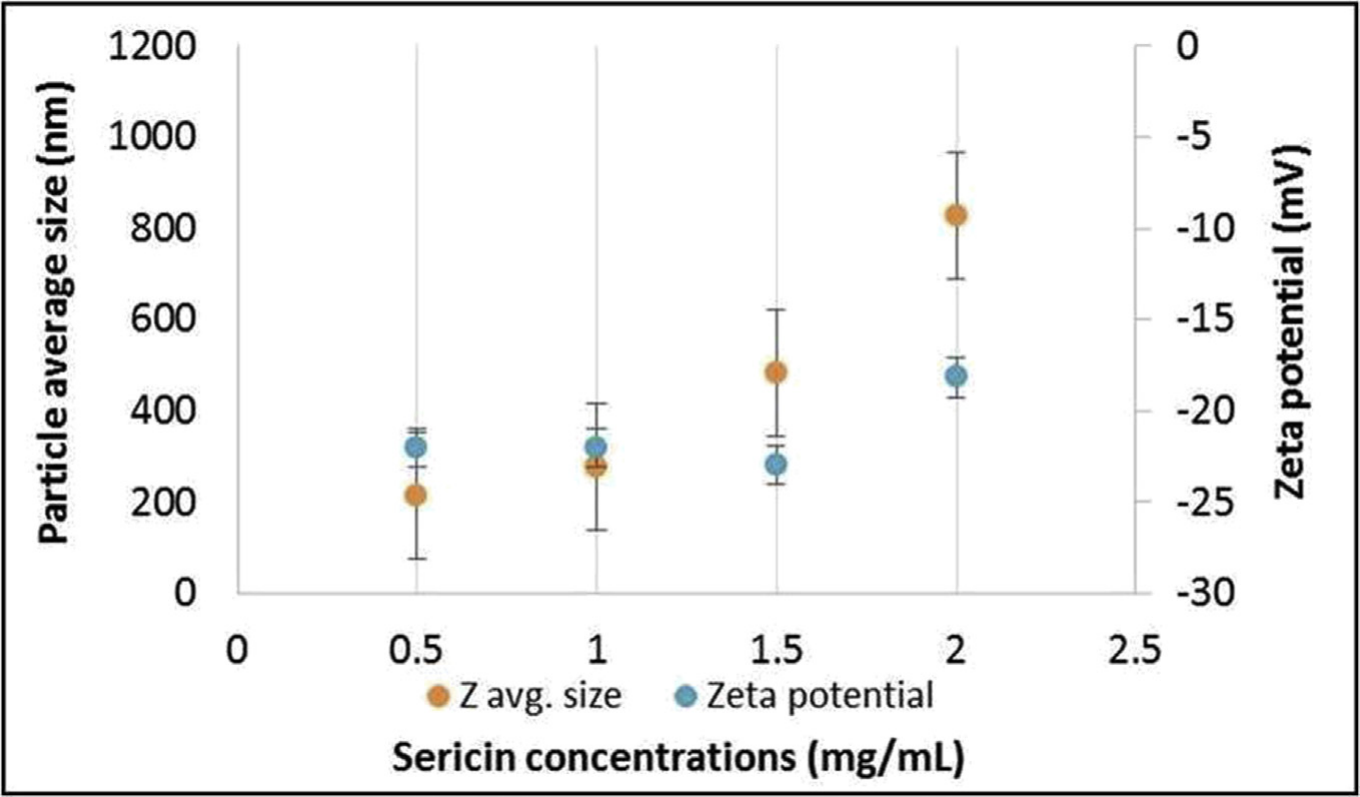
Hydrodynamic sizes and zeta potentials of sericin nanoparticle suspensions at varying sericin concentrations.

**Fig. 8:**
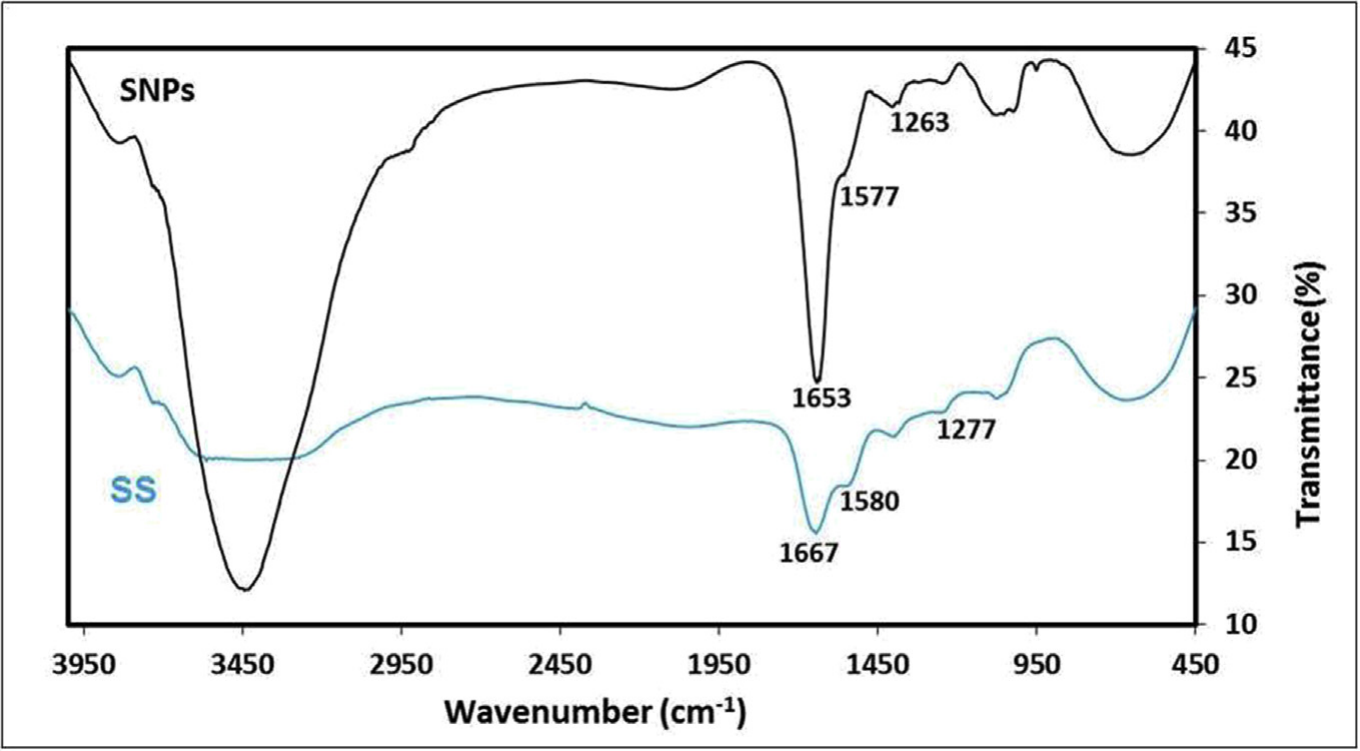
FTIR analysis of silk sericin (SS) and sericin nanoparticles (SNP).

**Fig. 9:**
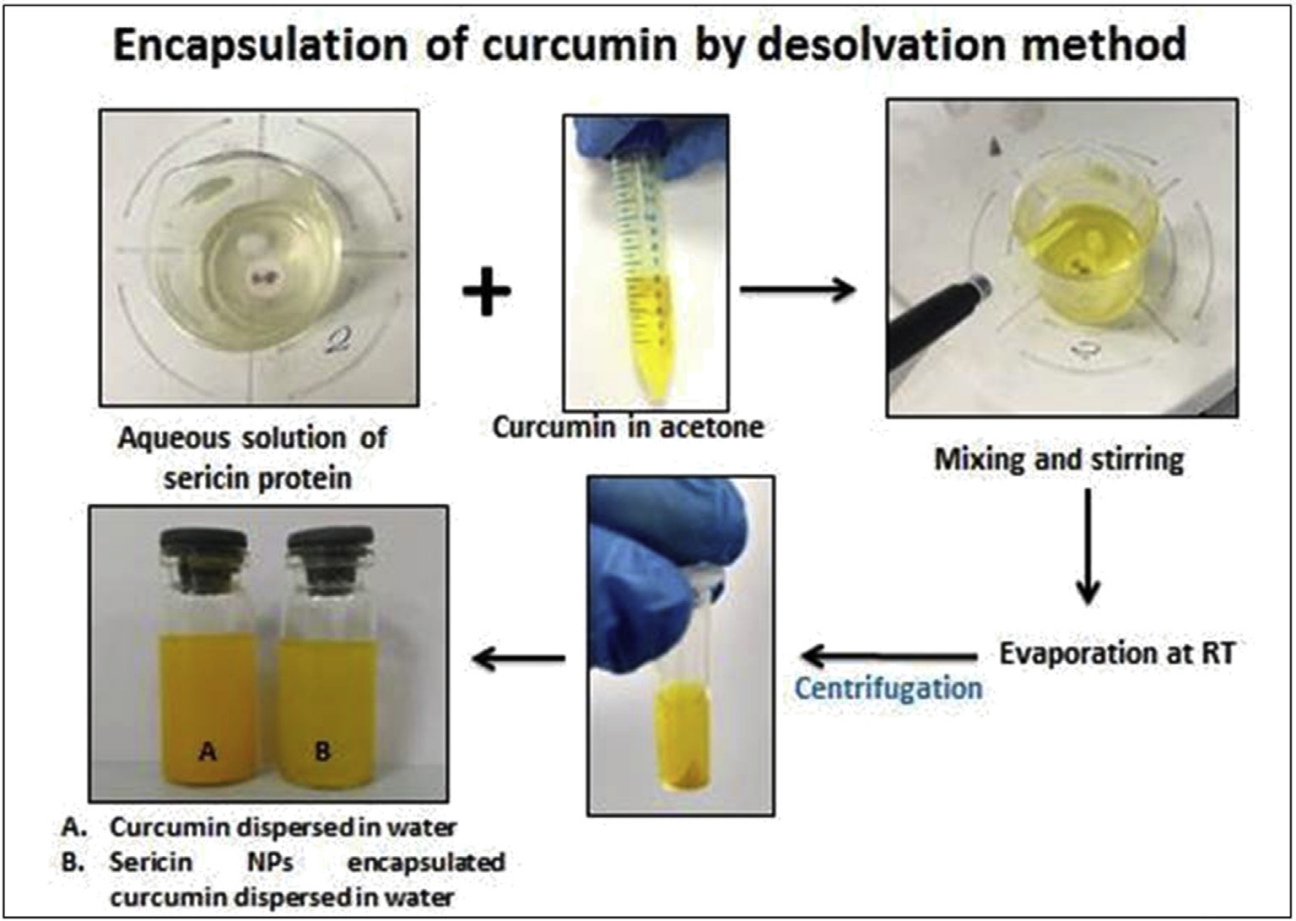
Encapsulation of curcumin in sericin nanoparticles by desolvation method.

Sericin protein is a waste product of silk industry and about 25–30% of waste sericin is generated during degumming process of silk [1]. The sericin protein used was extracted from *P. ricini* cocoons employing the high temperature and high pressure (HTHP) method (Fig. 1a–c). The molecular weight distribution of the extracted sericin was investigated using sodium dodecyl sulfate polyacrylamide gel electrophoresis (SDS-PAGE) analysis (Fig. 2). Synthesis of blank sericin nanoparticles was carried out by desolvation method using acetone as the desolvating agent. The physicochemical characteristics of the synthesized sericin nanoparticles were investigated through instrumental analyses *viz*, SEM (Fig. 3, Fig. 4, Fig. 5, Fig. 6), Zetasizer (Fig. 7) and FTIR (Fig. 8). Curcumin was used as the model hydrophobic drug for investigating the applicability of the sericin nanoparticles as nano drug carrier. Curcumin encapsulated nanoparticles were prepared by desolvation method with varying concentrations of curcumin solubilized in acetone of (Fig. 9). The encapsulation efficiency (EE %) and loading capacity (LC %) were calculated using standard methods (Table 3, Table 4).

## 2 Experimental design, materials, and methods

### 2.1 Extraction of sericin protein from silkworm cocoons

The autoclave method or high temperature and high pressure (HTHP) method of degumming was used for sericin extraction from Eri silkworm cocoons [3]. The cocoons were manually cleaned and cut into smaller pieces and autoclaved (60 mins, 15 psi, 121 ± 1 °C) at 1:50 ratio of sericin to water (*w/v*). The autoclaved solution containing sericin was filtered to remove any debris or stray fibers through Whatman filter paper no. 42 (Fig. 1a). The filtrate was lyophilized to obtain pure sericin powder and stored at 4 °C until further use (Table 1).

**Table 1:** Sericin extraction details.

Dry weight of cocoons (g)	Dilution factor (cocoons:water)	Dry weight of cocoons after autoclaving (g)	Weight of lyophilized sericin (g)	Extraction efficiency (%)	Yield percentage (%)
10	50x	8.86	0.22	11.4	19.3

### 2.2 Molecular weight distribution (MWD) of sericin

The MWD of the extracted sericin was investigated for fractionation due to the HTHP method of sericin extraction by SDS-PAGE [4] (Fig. 2).

### 2.3 Preparation of sericin nanoparticles

Sericin nanoparticles were synthesized by a one-step desolvation method using acetone as the desolvating agent [5]. 20 mL of varying concentrations of sericin solution (0.5, 1.0, 1.5 and 2.0 mg mL^−1^) was prepared to which 6 mL of acetone (30% v/v) was added dropwise while on constant stirring at 700–800 rpm. The suspension was kept under stirring condition for about 6 hours to volatilize the acetone. The sericin nanoparticles were collected and washed by centrifugation and used for further experiments.

### 2.4 Physicochemical characterization of blank and curcumin encapsulated sericin nanoparticles

The physicochemical characterization of blank and curcumin encapsulated sericin nanoparticles were carried out by different instrumental analyses. Morphological details (size, shape and aggregation) of the blank and encapsulated sericin nanoparticles were investigated by scanning electron microscopy (SEM, ZEISS Evo MA 10) (Fig. 3, Fig. 4, Fig. 5, Fig. 6). The SEM samples for both the blank and curcumin encapsulated sericin nanoparticles were prepared according to standard procedures [6]. UV–Vis spectrophotometry confirmed presence of in the curcumin encapsulated nanoparticle suspension. Zetasizer (Malvern, ZEN3690) was used to determine the hydrodynamic size range and surface charge (zeta potential) of the blank sericin nanoparticles (Table 2, Fig. 7). Fourier transform infrared (FTIR) analysis of sericin monomer and blank sericin nanoparticles was performed (FTIR, Thermo Scientific, Nicolet 6700 FT-IR) under attenuated total reflection (ATR) mode as described in literature [7] (Fig. 8).

**Table 2:** Zeta data of size and zeta potential of sericin nanoparticles.

Sericin concentration (mg/mL)	Zeta avg. size (nm)	Poly dispersity Index (PDI)	Zeta potential (mV)
0.5	213.1 ± 19.36	0.61 ± 0.12	−22.02 ± 1.15
1.0	278.15 ± 53	0.39 ± 0.21	−22.03 ± 1.19
1.5	484.13 ± 59	0.54 ± 0.09	−23.0 ± 3.59
2.0	829.41 ± 217	0.58 ± 0.11	−18.17 ± 1.38

### 2.5 Curcumin encapsulation

Curcumin encapsulation was done with a constant sericin concentration of 1.0 mg mL^−1^and varying concentrations of curcumin *viz.* 25 μM, 50 μM, 100 μM, 200 μM, 400 μM by desolvation method using acetone as the desolvating agent (Fig. 9).

### 2.6 Encapsulation efficiency and loading capacity

Encapsulation efficiency (EE %) and Loading capacity (LC %) were calculated (μg/mg) using standard methods [8], [9] (Table 3 and Table 4).

**Table 3:** Curcumin encapsulation efficiency at different concentrations of curcumin.

Added curcumin (μg)	Free curcumin (μg)	Encapsulated amount (μg)	Encapsulation efficiency (%)
9.2	5.60	3.6	39.13
18.4	7.63	10.77	58.53
36.8	11.02	25.78	70.05
73.6	10.85	62.75	85.25
147.2	22.45	124.75	84.75

**Table 4:** Loading capacity of sericin nanoparticles.

Weight of nanoparticles (mg)	Weight of encapsulated curcumin (mg)	Loading capacity (%)
12.63	0.0036	0.028
12.52	0.01077	0.086
12.43	0.02578	0.207
11.36	0.06275	0.5523
11.7	0.12475	1.066

### 2.7 Statistical analysis

Data are presented as mean ± SD values of the three independent experiments conducted in triplicates.
